# Apolipoprotein E decreases tau kinases and phospho-tau levels in primary neurons

**DOI:** 10.1186/1750-1326-1-18

**Published:** 2006-12-13

**Authors:** Hyang-Sook Hoe, Jacob Freeman, G William Rebeck

**Affiliations:** 1 Department of Neuroscience, Georgetown University, 3970 Reservoir Road NW, Washington, DC 20057-1464, USA

## Abstract

Apolipoprotein E (apoE) receptors act as signaling molecules in neurons, altering phosphorylation of numerous proteins after extracellular ligand binding and affecting neurite outgrowth, synapse formation, and neuronal migration. Since apoE is important in the pathogenesis of Alzheimer's disease (AD), we tested whether apoE treatment of neurons affected molecules important to phosphorylation of tau, such as GSK 3β, P35, and CDK5, and the phosphorylation of tau itself. Treatment of primary neurons with 2 uM apoE (or an apoE-derived peptide) decreased levels of phospho-GSK 3β, P35 and CDK5, and decreased levels of phosphorylated forms of tau. A lower concentration of apoE (100 nM) had no effect on these molecules. The alteration of tau phosphorylation by apoE was blocked by an inhibitor of the low-density lipoprotein receptor family, demonstrating the effects were due to receptor interactions. These results demonstrate that apoE affects several downstream signaling cascades in neurons: decreased tau kinases phosphorylation and inhibition of tau phosphorylation at Thr171 and Ser202/Thr205 epitopes. We conclude that apoE can alter levels of tau kinases and phospho-tau epitopes, potentially affecting tau neuropathological changes seen in AD brains.

## Introduction

Alzheimer's disease (AD) is defined neuropathologically by the presence of two types of protein aggregates: extracellular senile plaques, which are composed of the Aβ peptide, and intraneuronal neurofibrillary tangles (NFT), which are composed of phosphorylated forms of the tau protein [1-3]. Tau is a microtubule-associated protein with multiple phosphorylation sites [4]; hyperphosphorylation of tau in the AD brain is potentially promoted by several kinases, including GSK 3β, CDK5, and MARK [5]. Much AD-related research focuses on identifying factors that affect these neuropathological lesions and the risk of AD. One genetic factor that has been identified is the APOE genotype [6]. The APOE e4 allele is associated with increased Aβ deposition in brain [7-9]; evidence on whether APOE genotype also affects the accumulation of neurofibrillary tangles is more mixed [10].

The apoE protein is associated with high-density lipoproteins in the CNS [11], and is increased after several types of brain damage [12,13]. ApoE-lipoproteins bind members of the low-density lipoprotein (LDL) receptor family [14], receptors with complex ligand binding domains that allow interactions with large numbers of ligands. These receptors mediate uptake of apoE-containing lipoproteins, suggesting that apoE receptors could be important in the clearance of lipids after damage [15]. But stimulation of these receptors by ligands also mediates various neuronal signaling mechanisms. Binding of Reelin to LDL receptor family members promotes phosphorylation of the cytoplasmic disabled protein (Dab1) [16], and induces activation of Src and PKB kinases [17,18]. These processes are necessary for correct neuronal migration during development. Furthermore, Reelin inhibits phosphorylation of GSK 3β, but does not affect the activity of CDK5 [19]. We have found that apoE binding to these receptors also promotes Dab phosphorylation and stimulates intracellular activation of Src and PKB kinase [20]; it is unknown whether apoE also affects activation of tau kinases, and this question was the basis for the present study. ApoE induces neurite outgrowth and microtubule stability [21,22], and several studies have suggested that apoE or apoE fragments can access the cytoplasmic compartment of cells and directly bind to tau [23] or induce NTF-like inclusions [24]. Because apoE affects intracellular kinases through binding to its receptors, we examined the effects of apoE signaling on the activation of tau kinases and the phosphorylation of tau in vitro, using full-length apoE, or an apoE peptide derived from the receptor-binding region of apoE. Our results in primary neurons show that apoE treatment inhibited tau kinases (e.g., P35, P-GSK3β, and CDK5) and tau phosphorylation. These data suggest that apoE could alter tau phosphorylation and thus potentially affect the accumulation of NFT in the AD brain.

## Experimental procedures

### Chemicals

Recombinant human apoE2, E3 and E4 were purchased from Oxford Biomedical Research. The apoE peptide (EP; sequence LRKLRKRLLLRKLRKRLL) was synthesized by Johns Hopkins University of Medicine (Biosynthesis and sequencing facility, Baltimore, MD). This peptide, containing a tandem repeat of the receptor binding domain of apoE, has the same signaling properties as full length apoE [20]. Poly-D-lysine (P-7280) and phosphatase inhibitor cocktails (P-2850 and P-5726) were purchased from Sigma (St Louis, MO). CytoTox-ONE™ homogeneous Membrane Integrity Assay (G7891) was purchased from Promega (Madison, WI).

### Antibodies

Antibodies against phospho-GSK-3β (pY216, diluted 1:1000) and total GSK-3β were from BD Transduction Laboratories. Mouse monoclonal antibodies against p35 (diluted 1:1000), CDK5 (1:1000) and β-actin (1:1000) were purchased from Santa Cruz Biotechnology and BD Pharmingen. To examine the phosphorylation of tau, the following antibodies were used: mouse monoclonal antibody 5E2 (Upstate Biotechnology, diluted 1:1000), which recognizes a phosphorylation-independent tau epitope and is used to measure total tau; anti Tau-1 (Chemicon International, diluted 1:1000), which recognizes an unphosphorylated tau epitope; pS396 (Biosource International, diluted 1:1000), AT8 (Innogenetics, diluted 1:1000) and AT270 (Innogenetics, diluted 1:1000), which recognize phosphorylated tau epitopes.

### Neuronal cultures

Primary mouse embryonic cortical neuron cultures were prepared from embryonic day 16 Swiss-Webster mice as previously described [25]. Briefly, mouse E16 embryos brain cortices were chopped and trypsinized for 10 min at 37°C. After trypsinization, 0.4 μg/ml trypsin inhibitor, 0.025% DNase and 12 mM MgSO_4 _were added and mixed until the tissue was thoroughly homogenized. Cells were then transferred to Neurobasal medium containing B27 serum supplement (Invitrogen), 1 mM glutamine, gentamycin and cytosine-β-D-arabinofuranoside (Ara-C, 5 μg/ml) (Sigma). Neurons were seeded on 50 μg/ml poly-D- lysine coated 12 well tissue culture plates at a density of 2 × 10 ^6 ^per well. Toxicity was monitored by LDH release using the CytoTox-ONE™ Assay. To measure the effects of apoE on tau proteins, primary neuronal cells were treated with buffer or apoE peptide for 2 or 12 hours. For extraction of cells in RIPA buffer, neurons were washed with ice-cold PBS containing 10 mM NaF, ice-cold RIPA buffer (10 mM Tris-HCl, pH 7.2, 150 mM NaCl, 1% Triton X-100, 1% deoxycholate, 5 mM EDTA, protease inhibitor and phosphatase inhibitor cocktails) was added, then cells were centrifuged at 10,000 rpm for 5 min and the supernatant was collected. For extraction of cells in SDS buffer, the pellet after centrifugation was incubated with SDS sample buffer (62.5 mM Tris-HCl, pH 6.8, 2% SDS, 25% glycerol, 0.01% Bromophenol Blue)(Bio-Rad) including DNase for 30 min at 37°C. Additionally, cells were extracted directly with SDS sample buffer including DNase and incubated for 30 min at 37°C. Phosphorylation of tau and total tau levels were then measured in the SDS buffer extract, RIPA buffer extract, or in the SDS buffer extract following RIPA buffer extraction.

### Immunoblotting

Proteins from cell extracts were separated under denatured and reduced conditions by Tris-glycine polyacrylamide gel electrophoresis. Separated proteins were transferred onto PVDF membrane at 170 mA for 2 hours and blocked with 5% nonfat dry milk or 4% BSA. The blots were incubated with antibodies at room temperature for 1 hour. Horseradish peroxidase-conjugated secondary antibody was visualized by ECL detection system and exposed to film. Films were scanned using a ScanMaker 9800XL densitometer (Microtek), and relative levels of bands on a film were compared using Jandel SigmaStat software.

### Statistical analysis

Experiments were repeated a minimum of four times unless otherwise noted. All data was analyzed using ANOVA with Graphpad Prism 4 software, using Tukey's Multiple Comparison test for post-hoc analyses with significance determined as P < 0.05. Descriptive statistics were calculated with StatView 4.1 and displayed as an expressed mean ± S.E.M.

## Results

### ApoE inhibits tau kinases in primary neurons

**Figure 1:**
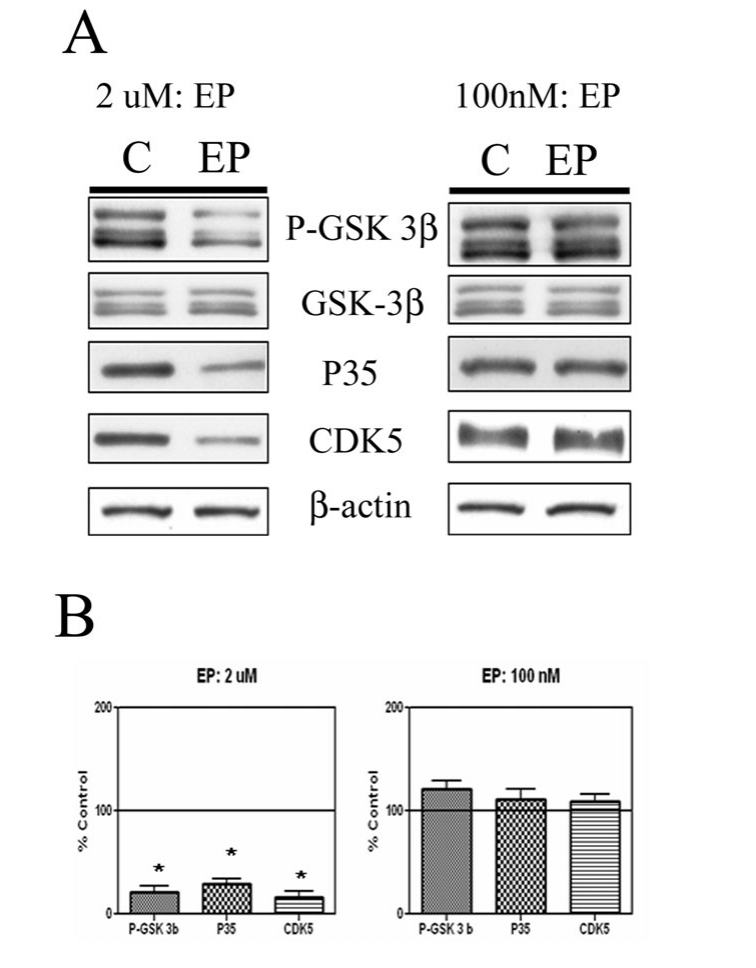
ApoE peptide (EP) affects tau kinases in primary neurons. Cultured primary neurons were treated with buffer or EP, a tandem repeat of the receptor-binding domain of apoE. Panel A: Cells were treated with control buffer ("C") or EP at 2 uM for 2 hours (left panels) or 100 nM overnight (right panels). Cell lysates (20 ug/lane) were electrophoresed and immunoblotted for phospho-GSK 3β (P-GSK 3β), total GSK 3β, P35, CDK5 and β-actin. β-Actin was measured from the same blots to ensure that equal protein was present in every lane. Panel B: Quantification of these blots revealed a significant decrease (*P < .05) in levels of P-GSK 3β(4 fold decrease), P35 (3.5 fold decrease), and CDK5 (5 fold decrease) with 2uM EP treatment. However, no significant decreases were noted with 100nM EP treatment. Histographs show mean +/- standard error, n = 4.

Since apoE altered activation of kinases including JNK, AKT and ERK1/2 in primary neurons [20], we tested whether apoE treatments also altered activation of several kinases potentially involved with phosphorylation of tau. We treated neurons with 2 uM apoE peptide for 2 hours, a treatment that increased AKT and ERK activation and decreased JNK activation [20]. This treatment decreased levels of P35, CDK5 and the phosphorylated form of GSK 3β (Fig. 1, left panel); no significant changes in levels of total GSK 3β or β-actin were observed. We did not observe production of P25, the active cleaved fragment of P35, under any conditions. Quantification of these blots revealed significant three to four fold decreases in total P35, total CDK5 and phospho-GSK 3β (Fig 1B, left panel). We also examined whether apoE concentrations normally found in CSF (approximately 100 nM [26]) altered levels of these kinases after 12 hours. This dose did not affect AKT, ERK, and JNK activation in vitro [20]. Similarly, apoE peptide at 100 nM for 12 hours did not change levels of phospho-GSK 3β, P35 and CDK5 (Fig. 1A, B, right panels). Thus, this apoE peptide induces reductions in various tau kinases, but only at concentrations above endogenous apoE levels.

**Figure 2:**
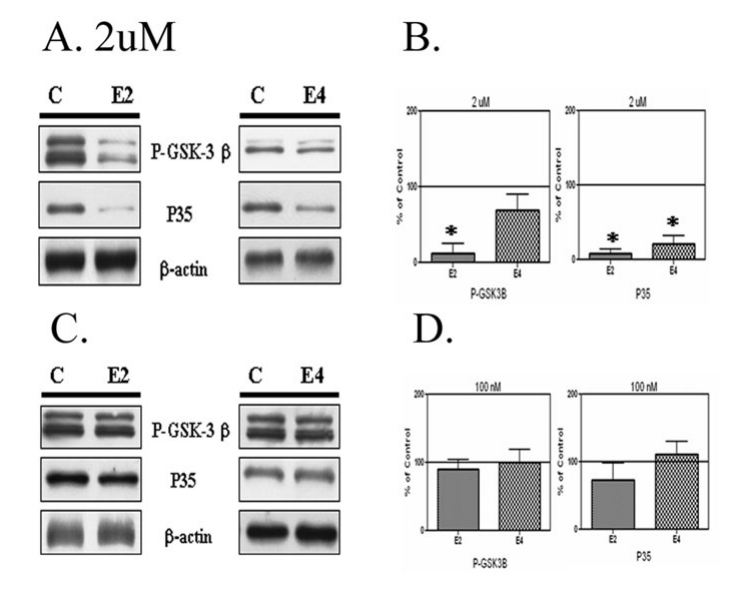
ApoE isoforms reduce phosphorylation of GSK 3β and P35. Panel A: Cells were treated with control buffer ("C"), apoE2 ("E2"), or apoE4 ("E4") at 2 uM for 2 hours. Cell lysates (20 ug/lane) were electrophoresed and immunoblotted for P-GSK 3β, P35, and β-actin. ApoE2 and E4 treatment resulted in decreased levels of P-GSK3β and P35. Panel B: ApoE2 caused the greatest decreases in GSK 3β phosphorylation, and apoE4 the least, with significant differences observed between each of the apoE isoforms. Both E2 and E4 caused significant decreases in P35, 96% and 79%, respectively. Panel C: Cells were treated with control buffer, E2, or E4 at 100 nM for 12 hours. Cell proteins were probed for P-GSK 3β, P35 and β-actin. Panel D: Neither apoE isoform at 100 nM had a significant effect on P-GSK3β and P35 levels.

We tested whether purified apoE isoforms also inhibited tau kinases. Exposure of cultured neurons to 2μM apoE2 or E4 decreased levels of phosphorylated GSK 3β and P35 (Fig. 2A). No significant changes in β-actin were observed after treatment with apoE isoforms (Fig. 2A). We quantified the changes in phospho-GSK 3β and P35; decreases in activation of GSK 3β (by 80% and 36 % for apoE2 and E4, respectively) were observed for the two apoE isoforms (Fig. 2B). ApoE2 and apoE4 also caused significant decreases in P35 (by 96% and 79%, respectively; Fig. 2B). We also examined whether apoE2 or apoE4 could affect these kinases at concentrations consistent with those observed in CSF (100 nM). We treated neurons with 100 nM apoE peptide for 12 hours, and found that these treatments did not change levels of phospho-GSK 3β (Fig. 2C, D). However, treatment of cells with 100 nM apoE2 slightly decreased levels of P35, by 23% (Fig. 2C, D), although apoE4 had no significant effects. No significant changes in β-actin were observed.

### ApoE decreases tau phosphorylation in primary neurons

**Figure 3:**
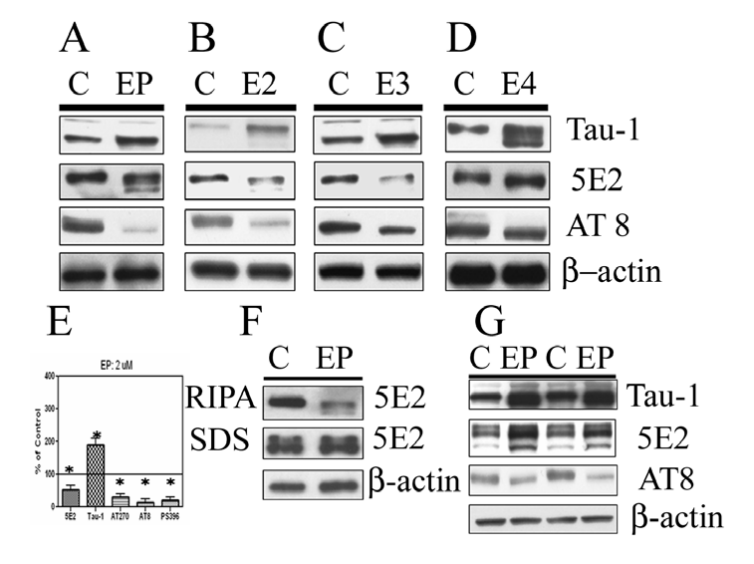
ApoE peptide and apoE isoforms affect phosphorylation of tau in primary neurons. Primary neurons were treated with control buffer ("C") or 2 uM EP (panel A), E2 (B), E3 (C) and E4 (D) for two hours. Cells were lysed in RIPA buffer (n = 4) and cell proteins (20 ug/lane) were separated by SDS-PAGE. Relative levels of tau epitopes were determined by immunoblotting with the indicated antibodies: anti-5E2, a measure of total tau; Tau 1, a measure of nonphosphorylated tau; pS396, AT8 and AT270, measures of phospho-tau epitopes. EP, E2, E3 resulted in increased Tau-1 immunoreactivity and decreased phosphorylation of tau at several sites. Quantification of these blots revealed that the apoE peptide induced a 115% increase in levels of unphosphorylated tau and a 70–90% decrease in phospho-tau proteins (panel E). Similar experiments with EP were conducted, but cells were extracted either RIPA buffer and sequentially in the stronger SDS buffer (panel F) or directly in the SDS buffer (panel G). A similar increase in non-phosphorylated tau and a decrease in phosphorylated tau were observed, but the levels of total tau were increased by EP treatment, in contrast to the decrease seen in panels A-D.

**Figure 5:**
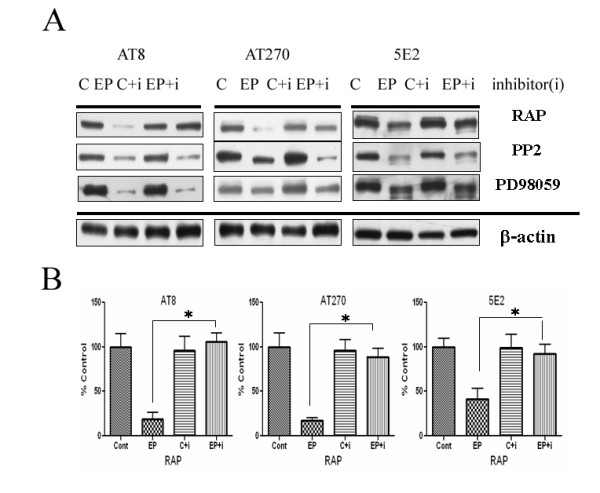
ApoE receptor inhibition alters tau phosphorylation. Primary neurons were pre-incubated in the presence of inhibitors ("i") (lanes 3 and 4) for 2h and then either exposed to control buffer ("C") (lanes 1 and 3) or 2 μM apoE peptide ("EP") (lane 2 and 4). Inhibitors used were RAP (1 μM), PP2 (1 μM), or PD98059 (10 μM). Cell extracts were was examined by immunoblots with AT8 (n = 4, left panel), AT270 (n = 4, middle panel) or 5E2(n = 4, right panel). RAP significantly reversed the effect of the apoE peptide on tau phosphorylation and total tau. PP2 and PD98059 treatments showed no significant changes. Panel B: Quantification of RAP blots (n = 4) demonstrated significant reversal of the effects that apoE (2uM) has on various tau and phospho-tau epitopes (p < 0.02).

We tested whether apoE treatments that decreased phospho-GSK 3β, P35, and CDK5 also decreased tau phosphorylations. We treated neurons for two hours with 2 uM apoE peptide, and extracted cell proteins in RIPA buffer. Levels of unphosphorylated tau, as measured by the Tau-1 antibody, were significantly increased by the apoE peptide (Fig. 3A). We blotted lysates with the anti-phospho-tau antibody AT8, which recognizes the phosphorylated residues of Ser202/Thr205 on the tau protein. ApoE peptide treatment significantly reduced levels of this phosphorylated form of tau (Fig. 3A). Similarly, anti-phospho-tau antibodies AT270 and PS396, which recognize the phosphorylated residues of Thr171 and Ser396 on the tau protein respectively, also showed significantly reduced levels in apoE peptide treated cells as compared to cells treated with buffer alone (data not shown, and Figure 5). Quantification of these blots revealed that the apoE peptide induced a 115% increase in levels of unphosphorylated tau and a 70–90% decrease in phospho-tau proteins (Fig 3E).

Surprisingly, we found that the levels of total tau were also significantly altered by the treatments with apoE peptide in this short (2 hour) period. Immunoblots with the antibody 5E2 (which recognizes tau irrespective of its phosphorylation state) demonstrated that levels of tau in cell extracts decreased by 69% (Figure 3A). We were concerned that all tau species were efficiently extracted in the RIPA buffer used, given that tau is microtubule-associated and thus might remain associated with cell debris after extraction. Therefore, we repeated the experiments, extracting the cells in RIPA buffer, and then extracting the insoluble fractions in SDS buffer (after treatment with DNase to decrease the viscosity of the extracts). Under these conditions, we did not observe significant decrease of total tau after EP treatment (Fig 3F), suggesting that apoE affected the proportion of total tau solubilized by RIPA, and apoE did not decrease the total levels of tau. As a further test of this hypothesis, we also extracted cells directly into the stronger buffer containing SDS. We found that the total level of tau observed was not decreased, but apparently increased after apoE peptide treatment (Figure 3G). We also found that the level of unphosphorylated tau (Tau-1) was still increased and the level of phospho-tau (AT8) was still decreased (Figure 3G) in the SDS extraction buffer. Thus, the apoE peptide treatment caused both a change in the phosphorylation state of tau, as well as a change in the distribution of the tau protein to alter its extraction in different buffers.

We tested whether similar changes occurred after treatment of cells with each of the full-length apoE isoforms. Each of the three apoE isoforms caused a decrease in the levels of phosphorylated tau (Fig. 3B, C, D). No significant changes in β-actin were observed. Interestingly, quantification of these blots revealed that apoE4 had the smallest effect of the three isoforms on tau phosphorylation state: it slightly increased levels of unphosphorylated tau (21%) and decreased phospho-tau proteins (35%).

**Figure 4:**
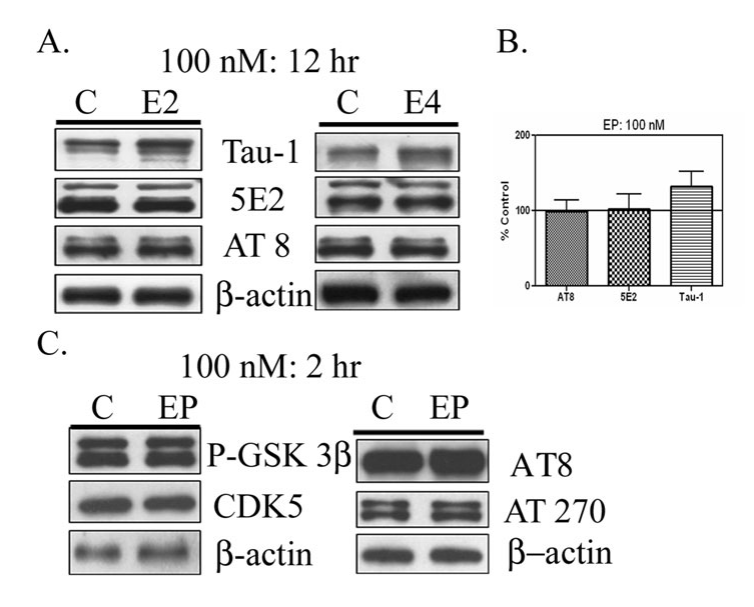
Panel A: Primary neurons were treated with control buffer ("C") or 100 nM apoE2 (left panel) and E4 (right panel) for 12 hours. Cells were lysed in RIPA buffer (n = 4) and cell proteins (20 ug/lane) were separated by SDS-PAGE. Relative levels of tau epitopes were determined by immunoblotting with 5E2, Tau-1, and AT8 antibodies. Neither apoE2 (100nM) nor E4 (100nM) significantly changed the levels of tau phosphorylation compared with control. No significant changes in β-actin were observed. Panel B: Quantification of these blots revealed no significant changes in 5E2, Tau-1, and AT8. N = 4 for all blots listed above and their quantification averages are what is depicted in the figure. Panel C: Primary neurons were treated with control buffer ("C") or 100 nM EP for two hours, and cellular proteins analyzed by immunoblot for phospho-GSK-3β, CDK5, AT8, AT270 and β-actin as above.

We also examined whether the lower dose of apoE (100 nM) inhibited tau phosphorylation. We found that treatment of cells with 100 nM apoE2 (Fig 4A, left panel) or 100 nM apoE4 (Fig 4A, right panel) did not change levels of tau phosphorylation compared with control (Fig 4A) after 12 hour treatments. No significant changes in β-actin were observed. Quantification of these blots revealed no significant changes in AT270, AT8 and PS396 (Fig 4B). We tested whether the lower dose of EP had an effect after the shorter period of time used in Figures 1–3 for the higher dose of EP. We observed no changes in levels of kinases, phospho-GSK-3β or CDK5 (Figure 4C). We also observed no differences in the levels of phospho-tau, as measured by the AT8 or AT270 antibodies (Figure 4C). Thus, the effects of apoE on tau kinases and tau phosphorylation only occurred after treatments with high levels of apoE or apoE peptide.

### Reduction in tau phosphorylation depends on apoE-receptor interaction

In order to test whether the observed changes to tau phosphorylation in primary neurons were mediated by members of LDL receptor family or were some direct effect of apoE, we pre-treated cells with RAP, an inhibitor of these receptors. RAP causes a prolonged redistribution of apoE receptors from dendritic to somatic compartments, potentially preventing signaling by treatments with subsequent ligands [25]. RAP treatment alone did not affect levels of phospho-tau epitopes (Fig. 5A, B). Treatment of neurons with the apoE peptide caused the significant decrease in phospho-taus (Thr 171, Ser202/Thr205), but these decreases were inhibited by pretreatment of cells with RAP (Fig. 5A, B, first row). The Src kinase inhibitor PP2 and the ERK kinase inhibitor PD98059 did not block the decrease in tau phosphorylation by apoE peptide (Fig. 5A, second and third row). No significant changes in β-actin were observed. These data suggest that decreases in phosphorylation of tau required binding of the apoE peptide to LDL receptor family members, but did not involve ERK or Src kinase cascades.

## Discussion

We have investigated how apoE signaling affects tau kinase activity and tau phosphorylation in primary neurons. In primary neurons, treatment of cells with 2 uM apoE (but not 100 nM) inhibited phosphorylation of GSK 3β, and decreased levels of P35 and CDK5 (Fig. 1). ApoE also decreased the levels of phosphorylated tau and increased the levels of unphosphorylated tau (Fig. 3). These treatments may also have altered the distribution of tau in the neurons, since increased levels of tau (but decreased levels of phosphorylated tau) were recovered in easily solubilized cell fractions (Fig. 3). These effects were mediated by members of the LDL receptor family, since their effects were blocked by the inhibitor RAP (Fig. 5).

Although apoE receptors were initially thought to function to endocytose ligands, several other functions of ligand binding have now been described. ApoE receptors transduce signals important for neuronal migration during development [27]. Ligand interactions with apoE receptors result in transient changes in Src, ERK and JNK phosphorylation [20,28]. ApoER2 interactions with NMDA receptors are modulated by ligand binding [29]. In glia, apoE receptors mediate anti-inflammatory effects of apoE [30]. Finally, in several cell types, ligand binding to apoE receptors increases the surface cleavage of the receptors [31]. Ligands for this family of receptors differ somewhat in whether they are freely able to be endocytosed (such as apoE), or whether they are attached to other cells or to the extracellular matrix (such as reelin). It will be interesting to determine whether there are differences in the signaling effects of ligands depending on whether their metabolism depends on endocytosis by apoE receptors.

These results complement studies of mouse knock-out models. Brains of APOE knock-out mice have increased levels of tau phosphorylation compared to wild-type controls [32,33]. Similarly, brains of Reeler mice, ApoER2-/-/VLDLr-/- mice, and Dab-1 knock-out mice have hyperphosphorylated tau [34]. Thus, chronic interruption of the apoE signaling pathway leads to increased tau phosphorylation in brain. Acute treatment of neurons with reelin caused decreased GSK-3β activation [16], and we report that treatment with apoE also caused decreased GSK-3β activation as well as reduced tau phosphorylation. Thus the acute effects of apoE and reelin are similar to each other, and consistent with the effects of chronic absence of apoE and reelin in vivo.

The fact that apoE affects the phosphorylation of the microtubule-associated protein tau is supportive of a function of apoE in promoting neurite outgrowth [25,35,36]. ApoE levels are induced after several types of brain damage [12,13], presumably as a mechanism for clearance of damaged membranes [37]. However, this apoE could also act as a signal for neurons to stabilize new neurites as a mechanism for regeneration after damage [38]. ApoE could alter the stability of the microtubules by altering tau and phospho-tau levels, allowing permanent changes to the neuronal cytoskeleton that need to accompany neurite outgrowth.

In the AD brain, the APOE genotype affects levels of Aβ deposition [7,8], and apoE is a component of most plaques [39,40]. In fact, because of its strong affinity for Aβ, apoE has been referred to as a pathological chaperone [41]. The presence of the apoE receptor binding domain on amyloid deposits [40] suggests that apoE on plaques could provide chronic signals via apoE receptors to neurons in the vicinity of plaques. These signals could include increased phosphorylation of substrates of kinases such as ERK and Src [20] or decreased phosphorylation of substrates of kinases such as JNK [20], GSK 3β and CDK5 (Fig 1). Since kinases in both groups have been linked to increased tau phosphorylation (e.g. ERK [42] and GSK-3β [43]), apoE presence on plaques could contribute both to the formation and the inhibition of phospho-tau-positive dystrophic neurites found around neuritic plaques. We previously have found different strengths of the signaling effects for the apoE isoforms. ApoE2 had the most effect on ERK activation and JNK inhibition [20] and apoE4 the least; a similar pattern was observed for the different apoE isoforms on receptor cleavage [31]. In this study, apoE2 decreased levels of phospho-GSK-3β and CDK5 more than apoE4 (Figure 2). If the acute effects of apoE on neurons applied to more chronic exposures, apoE2 on plaques would be expected to inhibit phospho-tau levels the most, and apoE4 would be expected to have the least effect. Several studies have indicated that higher levels of phospho-tau in AD brain are associated with apoE4 than with apoE3 or apoE2 [42], correlating with the in vitro effects of these isoforms on GSK-3β and CDK5 inhibition (and not with their effects on ERK activation).

Animal models have also implicated apoE in disruption of neuronal cytoskeleton. Neuronal expression of apoE4 is associated with prominent axonopathy [44] and increased tau phosphorylation [42,45]. These processes may involve intraneuronal proteolysis of apoE [45]. Expression of APOE is glial under most conditions, but studies of the APOE promoter demonstrate that under some kainic acid treatment condition [46], APOE may be expressed in neurons. Under some conditions, apoE may be present within the neuronal cytoplasm [21], where more direct effects on tau and microtubules are possible. ApoE directly inhibits GSK-3β phosphorylation of tau [47] and apoE alters the pattern of tau phosphorylation by GSK-3β [43]. These effects of apoE would not depend on interactions with apoE receptors, as we observed here (Figure 5).

In this study, we report that apoE can decrease levels of tau kinases and phospho-tau proteins via extracellular interactions with apoE receptors. These effects of apoE could be important for the strong association of the APOE gene with risk of AD.

## References

[B1] Gotz J, Chen F, van Dorpe J, Nitsch RM (2001). Formation of neurofibrillary tangles in P301l tau transgenic mice induced by Abeta 42 fibrils. Science.

[B2] Mandelkow EM, Mandelkow E (1994). Tau protein and Alzheimer's disease. Neurobiol Aging.

[B3] Mandelkow E (1999). Alzheimer's disease. The tangled tale of tau. Nature.

[B4] Genis I, Gordon I, Sehayek E, Michaelson DM (1995). Phosphorylation of tau in apolipoprotein E-deficient mice. Neurosci Lett.

[B5] Morishima-Kawashima M, Hasegawa M, Takio K, Suzuki M, Yoshida H, Titani K, Ihara Y (1995). Proline-directed and non-proline-directed phosphorylation of PHF-tau. J Biol Chem.

[B6] Strittmatter WJ, Roses AD (1995). Apolipoprotein E and Alzheimer disease. Proc Natl Acad Sci U S A.

[B7] Schmechel DE, Saunders AM, Strittmatter WJ, Crain BJ, Hulette CM, Joo SH, Pericak-Vance MA, Goldgaber D, Roses AD (1993). Increased amyloid beta-peptide deposition in cerebral cortex as a consequence of apolipoprotein E genotype in late-onset Alzheimer disease. Proc Natl Acad Sci U S A.

[B8] Rebeck GW, Reiter JS, Strickland DK, Hyman BT (1993). Apolipoprotein E in sporadic Alzheimer's disease: allelic variation and receptor interactions. Neuron.

[B9] Strittmatter WJ, Burke JR, DeSerrano VS, Huang DY, Matthew W, Saunders AM, Scott BL, Vance JM, Weisgraber KH, Roses AD (1996). Protein: protein interactions in Alzheimer's disease and the CAG triplet repeat diseases. Cold Spring Harb Symp Quant Biol.

[B10] Nagy Z, Esiri MM, Jobst KA, Johnston C, Litchfield S, Sim E, Smith AD (1995). Influence of the apolipoprotein E genotype on amyloid deposition and neurofibrillary tangle formation in Alzheimer's disease.. Neuroscience.

[B11] Pitas RE, Boyles JK, Lee SH, Hui D, Weisgraber KH (1987). Lipoproteins and their receptors in the central nervous system. Characterization of the lipoproteins in cerebrospinal fluid and identification of apolipoprotein B,E(LDL) receptors in the brain.. J Biol Chem.

[B12] Poirier J, Hess M, May PC, Finch CE (1991). Cloning of hippocampal poly(A) RNA sequences that increase after entorhinal cortex lesion in adult rat. Brain Res Mol Brain Res.

[B13] Page KJ, Hollister RD, Hyman BT (1998). Dissociation of apolipoprotein and apolipoprotein receptor response to lesion in the rat brain: an in situ hybridization study. Neuroscience.

[B14] Herz J, Beffert U (2000). Apolipoprotein E receptors: linking brain development and Alzheimer's disease. Nat Rev Neurosci.

[B15] Fagan AM, Murphy BA, Patel SN, Kilbridge JF, Mobley WC, Bu G, Holtzman DM (1998). Evidence for normal aging of the septo-hippocampal cholinergic system in apoE (-/-) mice but impaired clearance of axonal degeneration products following injury.. Exp Neurol.

[B16] Beffert U, Morfini G, Bock HH, Reyna H, Brady ST, Herz J (2002). Reelin-mediated signaling locally regulates protein kinase B/Akt and glycogen synthase kinase 3beta. J Biol Chem.

[B17] Arnaud L, Ballif BA, Forster E, Cooper JA (2003). Fyn tyrosine kinase is a critical regulator of disabled-1 during brain development. Curr Biol.

[B18] Bock HH, Herz J (2003). Reelin activates SRC family tyrosine kinases in neurons. Curr Biol.

[B19] Hiesberger T, Trommsdorff M, Howell BW, Goffinet A, Murphy MC, Cooper JA, Herz J (1999). Direct binding of Reelin to VLDL receptor and ApoE receptor 2 induces tyrosine phosphorylation of disabled-1 and modulates tau phosphorylation.. Neuron.

[B20] Hoe HS, Harris DC, Rebeck GW (2005). Multiple pathways of apolipoprotein E signaling in primary neurons.. J Neurochem.

[B21] Strittmatter WJ, Saunders AM, Goedert M, Weisgraber KH, Dong LM, Jakes R, Huang DY, PericakVance M, Schmechel D, Roses AD (1994). Isoform-specific interactions of apolipoprotein E with microtubule-associated protein tau: implications for Alzheimer disease. Proc Natl Acad Sci U S A.

[B22] Nathan BP, Chang KC, Bellosta S, Brisch E, Ge N, Mahley RW, Pitas RE (1995). The inhibitory effect of apolipoprotein E4 on neurite outgrowth is associated with microtubule depolymerization.. J Biol Chem.

[B23] Strittmatter WJ, Bova Hill C (2002). Molecular biology of apolipoprotein E. Curr Opin Lipidol.

[B24] Huang Y, Liu XQ, Wyss-Coray T, Brecht WJ, Sanan DA, Mahley RW (2001). Apolipoprotein E fragments present in Alzheimer's disease brains induce neurofibrillary tangle-like intracellular inclusions in neurons. Proc Natl Acad Sci U S A.

[B25] Qiu Z, Hyman BT, Rebeck GW (2004). Apolipoprotein E receptors mediate neurite outgrowth through activation of p44/42 mitogen-activated protein kinase in primary neurons. J Biol Chem.

[B26] Han X, Cheng H, Fryer JD, Fagan AM, Holtzman DM (2003). Novel Role for Apolipoprotein E in the Central Nervous system. J Biol Chem.

[B27] Trommsdorff M, Gotthardt M, Hiesberger T, Shelton J, Stockinger W, Nimpf J, Hammer RE, Richardson JA, Herz J (1999). Reeler/Disabled-like disruption of neuronal migration in knockout mice lacking the VLDL receptor and ApoE receptor 2.. Cell.

[B28] Ohkubo N, Mitsuda N, Tamatani M, Yamaguchi A, Lee YD, Ogihara T, Vitek MP, Tohyama M (2001). Apolipoprotein E4 stimulates cAMP response element-binding protein transcriptional activity through the extracellular signal-regulated kinase pathway.. J Biol Chem.

[B29] Hoe HS, Pocivavsek A, Chakraborty G, Fu Z, Vicini S, Ehlers MD, Rebeck GW (2006). Apolipoprotein E receptor 2 interactions with the N-methyl-D-aspartate receptor.. J Biol Chem.

[B30] Ladu MJ, Shah JA, Reardon CA, Getz GS, Bu G, Hu J, Guo L, Van Eldik LJ (2001). Apolipoprotein E and apolipoprotein E receptors modulate A beta-induced glial neuroinflammatory responses.. Neurochem Int.

[B31] Hoe HS, Rebeck GW (2005). Regulation of ApoE receptor proteolysis by ligand binding.. Mol Brain Res.

[B32] Genis L, Chen Y, Shohami E, Michaelson DM (2000). Tau hyperphosphorylation in apolipoprotein E-deficient and control mice after closed head injury. J Neurosci Res.

[B33] Cedazo-Minguez A, Popescu BO, Blanco-Millan JM, Akterin S, Pei JJ, Winblad B, Cowburn RF (2003). Apolipoprotein E and beta-amyloid (1-42) regulation of glycogen synthase kinase-3beta. J Neurochem.

[B34] Brich J, Shie FS, Howell BW, Li R, Tus K, Wakeland EK, Jin LW, Mumby M, Churchill G, Herz J, Cooper JA (2003). Genetic modulation of tau phosphorylation in the mouse. J Neurosci.

[B35] Fagan AM, Bu G, Sun Y, Daugherty A, Holtzman DM (1996). Apolipoprotein E-containing high density lipoprotein promotes neurite outgrowth and is a ligand for the low density lipoprotein receptor-related protein. J Biol Chem.

[B36] Nathan BP, Bellosta S, Sanan DA, Weisgraber KH, Mahley RW, Pitas RE (1994). Differential effects of apolipoproteins E3 and E4 on neuronal growth in vitro. Science.

[B37] Poirier J (1996). Apolipoprotein E in the brain and its role in Alzheimer's disease. J Psychiatry Neurosci.

[B38] Teter B, Ashford JW (2002). Neuroplasticity in Alzheimer's disease. J Neurosci Res.

[B39] Namba Y, Tomonaga M, Kawasaki H, Otomo E, Ikeda K (1991). Apolipoprotein E immunoreactivity in cerebral amyloid deposits and neurofibrillary tangles in Alzheimer's disease and kuru plaque amyloid in Creutzfeldt-Jakob disease. Brain Res.

[B40] Cho HS, Hyman BT, Greenberg SM, Rebeck GW (2001). Quantitation of apoE domains in Alzheimer disease brain suggests a role for apoE in Abeta aggregation. J Neuropathol Exp Neurol.

[B41] Wisniewski T, Frangione B (1992). Apolipoprotein E: a pathological chaperone protein in patients with cerebral and systemic amyloid. Neurosci Lett.

[B42] Harris FM, Brecht WJ, Xu Q, Mahley RW, Huang Y (2004). Increased tau phosphorylation in apolipoprotein E4 transgenic mice is associated with activation of extracellular signal-regulated kinase: modulation by zinc.. J Biol Chem.

[B43] Gibb GM, Pearce J, Betts JC, Lovestone S, Hoffmann MM, Maerz W, Blackstock WP, Anderton BH (2000). Differential effects of apolipoprotein E isoforms on phosphorylation at specific sites on tau by glycogen synthase kinase-3 beta identified by nano-electrospray mass spectrometry. FEBS Letters.

[B44] Tesseur I, Dorpe JV, Bruynseels K, Bronfman F, Sciot R, Lommel AV, Leuven FV (2000). Prominent Axonopathy and Disruption of Axonal Transport in Transgenic Mice Expressing Human Apolipoprotein E4 in Neurons of Brain and Spinal Cord.. Ameican Journal of Pathology.

[B45] Brecht WJ, Harris FM, Chang S, Tesseur I, Yu GQ, Xu Q, Fish JD, Wyss-Coray T, Buttini M, Mucke L, Mahley RW, Huang Y (2004). Neuron-specific Apolipoprotein E4 proteolysis is associated with increased Tau phosphorylation in brains of Transgenic mice.. J Neurosci.

[B46] Xu Q, Bernardo A, Walker D, Kanegawa T, Mahley RW, Huang Y (2006). Profile and regulation of apolipoprotein E (ApoE) expression in the CNS in mice with targeting of green fluorescent protein gene to the ApoE locus.. J Neurosci.

[B47] Flaherty D, Lu Q, Soria J, Wood JG (1999). Regulation of Tau Phosphorylation in Microtubule Fractions by Apolipoprotein E. J Neurosci Res.

